# Supplementary material for: Scaling quantum approximate optimization on near-term hardware
Source: Sci Rep. 2022 Jul 20;12:12388. doi: 10.1038/s41598-022-14767-w (PMC9300688; doi:10.1038/s41598-022-14767-w)
Supplement: Supplementary file 1 — Supplementary Information. [file 41598_2022_14767_MOESM1_ESM.pdf]

Supplemental Information: Scaling Quantum Approximate Optimization on Near-term Hardware

Phillip C. Lotshaw,[1,*] Thien Nguyen[†,2,3] Anthony Santana[‡,2] Alexander McCaskey[§,2,3]
Rebekah Herrman,[4] James Ostrowski,[4] George Siopsis,[5] and Travis S. Humble[1,3]

[1] *Quantum Computational Sciences Group, Oak Ridge National Laboratory, Oak Ridge, Tennessee 37830 USA*
[2] *Beyond Moore Computing Group, Oak Ridge National Laboratory, Oak Ridge, Tennessee 37830 USA*
[3] *Quantum Science Center, Oak Ridge National Laboratory, Oak Ridge, Tennessee 37830 USA*
[4] *Department of Industrial and Systems Engineering, University of Tennessee, Knoxville, Tennessee 37996-2315 USA*
[5] *Department of Physics and Astronomy, University of Tennessee, Knoxville, Tennessee 37996-1200 USA*

(Dated: July 5, 2022)

CIRCUIT MAPPING COMPUTATIONS

We map QAOA problem instances to hardware circuits using the Enfield software library [1] implemented within the XACC programming framework [2, 3]. We assessed performance of the circuit mapping algorithms WPM, CHW, BMT, and SABRE on example problems, finding that SABRE [4] gave superior performance and time-to-solution scaling with problem size. We therefore used SABRE for all of our circuit mappings. We optimized two adjustable parameters of the SABRE algorithm to minimize gate counts for each of our test sets. The first parameter "iterations" determines how many times SABRE generates random initial placements. Each of these placements is optimized by SABRE, which outputs the placement with the smallest depth as the final result. The second parameter used by SABRE is called "lookahead" and determines a balance between current and future gates in the objective function for varying steps in the algorithm, see Ref. [4] for details.

Figure S1 shows an example of the convergence behavior for the "iterations" and "lookahead" parameters for a series of five "shuffled" initializations, as described in detail in the next section, for the test-set of 3-regular graphs of size $n = 20$. For each initialization, the results show a clear dependence on the values of "iterations" and "lookahead", which become steady when both of these parameters are at least forty. We set each parameter equal to 40 to compute our final results for the test set. We observed similar behavior for these parameters at $n = 40$, with results that appeared convergent when iterations and lookahead are equal. For $n = 60$ we optimize the parameters assuming they are equal based on our results from $n = 20$ and $n = 40$. Table S1 lists all the final values we use for these parameters for each of our test sets.

n	graph ensemble	shuffles	Sabre iterations	lookahead
7	non-isomorphic	50	20	10
20	3-regular	50	40	40
40	3-regular	50	100	100
60	3-regular	50	140	140

TABLE S1: SABRE parameters.

Improvements to optimized circuit layouts

We improve the SABRE circuit layouts by implementing QAOA-specific cancellations of circuit elements. CNOT gates can be removed when a SWAP gate ($\mathrm{SWAP} = \mathrm{CNOT}_{ij}\mathrm{CNOT}_{ji}\mathrm{CNOT}_{ij}$) appears next to a trio of gates for a

* lotshawpc@ornl.gov;
† now at Quantum Brilliance
‡ now at Q-CTRL
§ now at NVIDIA

This manuscript has been authored by UT-Battelle, LLC under Contract No. DE-AC05-00OR22725 with the U.S. Department of Energy. The United States Government retains and the publisher, by accepting the article for publication, acknowledges that the United States Government retains a non-exclusive, paid-up, irrevocable, world-wide license to publish or reproduce the published form of this manuscript, or allow others to do so, for United States Government purposes. The Department of Energy will provide public access to these results of federally sponsored research in accordance with the DOE Public Access Plan. (http://energy.gov/downloads/doe-public-access-plan)

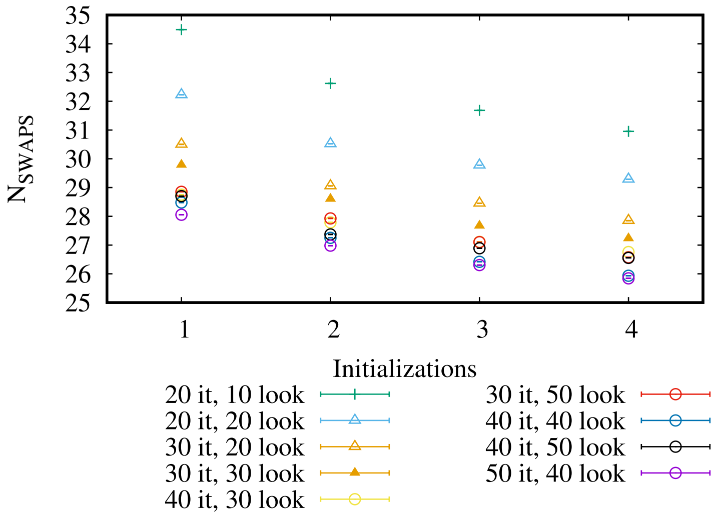

FIG. S1: Example convergence behavior of SABRE for variations in the "iterations" (its) and "lookahead" (look) parameters, for 3-regular graphs at $n = 20$

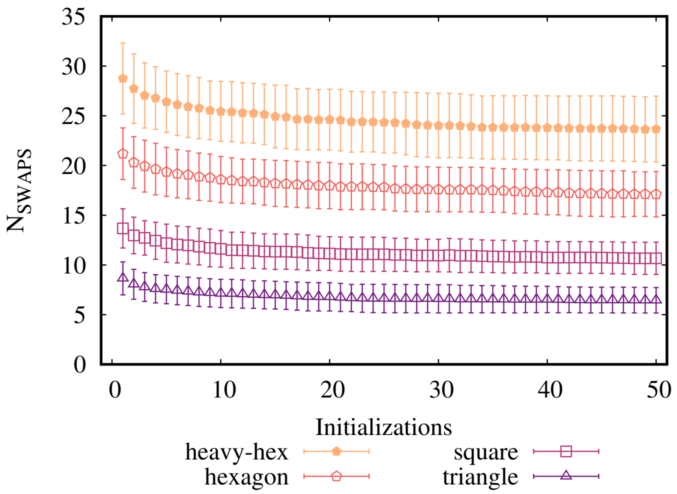

FIG. S2: Example convergence behavior with varying shuffled initializations at $n = 20$.

two-qubit cost term ($\exp(-i\gamma_l J_{i,j} Z_i Z_j) = \mathrm{CNOT}_{ij}\mathrm{R}_j(2J_{i,j}\gamma)\mathrm{CNOT}_{ij}$). This gives adjacent and identical CNOT_{ij} gates which can be removed since $\mathrm{CNOT}_{ij}\mathrm{CNOT}_{ij} = \mathbb{1}$. Although each SWAP gate is defined by a series of three CNOT gates, the net gate cost from adding a SWAP gate with a cancellation is only $\sigma = 1$ additional CNOT gate, since two CNOT gates are removed in the cancellation. To increase the number of these cancellations, we defined a "shuffling" algorithm that rearranges the commuting edge terms $\mathrm{CNOT}_{ij}\mathrm{R}_j(2J_{i,j}\gamma)\mathrm{CNOT}_{ij}$ in the circuit that we input to SABRE for optimization. We found this rearrangement also reduces the total number of SWAP gates by finding more efficient series of gates for the hardware.

We define a shuffling procedure that works in a loop outside SABRE to generate a random ordering for the commuting $\mathrm{CNOT}_{ij}\mathrm{R}_j(2J_{i,j}\gamma)\mathrm{CNOT}_{ij}$ gate-trios in the circuit. At each step in the loop, a shuffled QAOA instance is passed to SABRE to determine a final optimized hardware circuit, keeping the circuit with the fewest CNOT gates as the final optimized solution reported in the paper. Figure S2 shows the convergence behavior of SABRE with additional shuffling iterations. The changes between iterations are very small by about 50 iterations. We find similar behavior for all our test sets, with small changes between subsequent shuffling iterations around 50, hence we use 50 shuffles for all our results. Each of the 50 shuffle iterations is optimized by SABRE over a number of random qubit initial placements given by the "iterations" parameter from Table S1 to identify a final optimized instance. From the values in the table, this gives 1000-7000 optimizations per graph to identify a single best solution.

Algorithm 1: QAOA SABRE Heuristic Search

Input: Problem Graph, Topology (Qubit Coupling Graph), Number of Shuffles, SABRE iterations, lookahead
Output: Best Mapped Circuit, Best CNOT count
Ising_Ham ← Convert(Problem Graph)// problem graph edge list $< i, j >, < i', j' >, \ldots,$
// Ising_Ham ← $Z_i Z_j + Z_{i'} Z_{j'} + \ldots$
Shuffle_Trials ← 0
while Shuffle_Trials < Number of Shuffles **do**
  Reorders the Ising_Ham randomly // random ordering of the $Z_i Z_j, Z_{i'} Z_{j'}, \ldots$
  Circuit ← Generate_QAOA_Circuit(Ising_Ham) // gate ordering based on Ising_Ham
  (Mapped Circuit, Numer of SWAPs) ← SABRE(Circuit, Topology, SABRE iterations, lookahead)
    // inner loop in SABRE optimizes circuits for random initial placements = 1,. . . , SABRE iterations
  Optimized Circuit ← Optimize(Mapped Circuit)
    // remove adjacent $\mathrm{CNOT}_{i,j}\mathrm{CNOT}_{i,j}$, remove $\mathrm{CNOT}_{i,j}$ in first layer
  CNOT count ← Calculate_CNOT_count(Optimized Circuit)
  if CNOT count < Best CNOT count **then**
    Best CNOT count ← CNOT count
    Best Mapped Circuit ← Optimized Circuit
  end
  Shuffle_Trials++
end

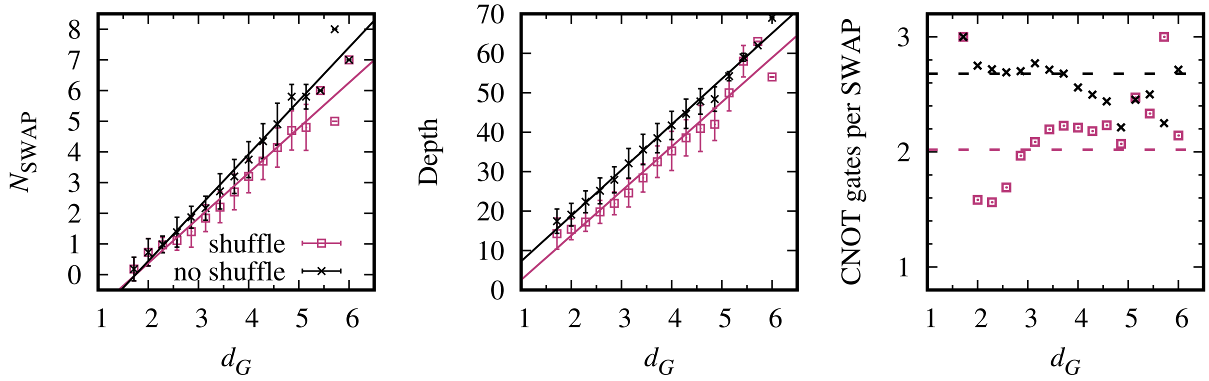

(a) (b) (c)

FIG. S3: SABRE optimization with and without an exterior loop that shuffles commuting two-qubit circuit components for the edges in the problem graph. (a) Shuffling decreases the number of SWAP gates, (b) the circuit depth, and (c) the net number of CNOT gates per SWAP gate σ after cancelling pairs $\mathrm{CNOT}_{ij}\mathrm{CNOT}_{ij}$ in the circuits.

A final improvement comes from cancelling the first layer of CNOT gates in the total QAOA circuit—the initial state is $\mathrm{H}^{\otimes n}|0\rangle^{\otimes n} = |+\rangle^{\otimes n}$ and $\mathrm{CNOT}_{ij}|+\rangle^{\otimes n} = |+\rangle^{\otimes n}$, hence the first layer of CNOT gates can be removed. This gives the factor N_0 in Eq. (5) of the main text. To systematically search for CNOT gates to cancel in the first layer, we traverse the circuit from left to right and record the operands of each CNOT gate. If both qubit operands have never been seen before, we cancel the CNOT gate. Note there is an optimal ordering of edge terms ($Z_i Z_j$) to maximize the number of CNOT gates that can be canceled in this way—for example, consecutive disjoint edge terms, such as $Z_0 Z_1$ and $Z_2 Z_3$, result in more gate cancellation opportunities than a chaining list of terms, such as $Z_0 Z_1$ and $Z_1 Z_2$. The procedure allows us to cancel at most $\lfloor n/2 \rfloor$ gates, as noted Majumdar *et al.* [5], since we can have at most $\lfloor n/2 \rfloor$ sets of $Z_i Z_j$ gates with disjoint sets of operands i, j.

Pseudocode describing the overall optimization approach is given in Algorithm 1. Figure S3 evaluates the effectiveness of the shuffling iterations relative to an equal number of calls to SABRE without shuffling, for the set of non-isomorphic graphs at $n = 7$ mapped to a square hardware grid. In each figure, the horizontal axis shows the average vertex degree d_G for the graphs, i.e., the average number of non-zero $J_{i,j} Z_i Z_j$ terms per qubit i. In the left and central figures, the shuffling algorithm is successful in decreasing the number of SWAP gates and the circuit depth, as demonstrated by the linear fits with parameters shown in Table S2. The rightmost figure shows the number of CNOT gates per SWAP gate σ, which is significantly decreased using the shuffling routine, especially at small

	a (shuffle)	b (shuffle)	a (no shuffle)	b (no shuffle)
N_{SWAP}	1.47 ± 0.07	-2.6 ± 0.3	1.74 ± 0.07	-3.0 ± 0.3
Depth	11.3 ± 0.6	-9 ± 3	11.6 ± 0.3	-4 ± 1

TABLE S2: Fit parameters from the linear fits $f(d) = ad_G + b$ in Fig. S3.

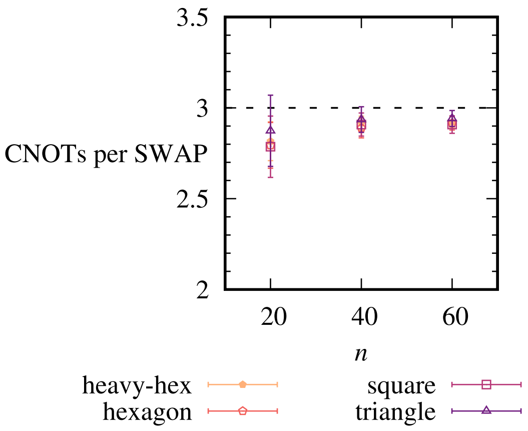

FIG. S4: Average increase in CNOT gate counts per SWAP gate σ in the final circuits for 3-regular graphs. Values $\sigma < 3$ come from cancelling adjacent pairs $\text{CNOT}_{ij}\text{CNOT}_{ij}$ in the circuits.

d. The horizontal dotted lines show the average numbers of CNOT gates per SWAP gate, averaged over all graphs with one or more SWAP gate. The average σ decreases by about 0.7 when the shuffling routine is implemented. Overall, the shuffling routine performs well at reducing the QAOA circuit cost by including QAOA-specific circuit commutativity in the SABRE optimization. Figure S4 evaluates σ for the sets of 3-regular graphs. These increase close to $\sigma = 3$ as the graph size n increases. We therefore use $\sigma = 3$ for 3-regular graphs at large n in our scaling analysis.

Degree-based SWAP Gate Lower Bound at Small n

We evaluate SABRE performance in comparison with a simple lower bound for the number of SWAP gates for the non-isomorphic graphs at $n = 7$. The number of SWAP gates can be bounded in terms of the degrees of the hardware connectivity graph and the graph for the cost Hamiltonian, i.e., the set of non-zero $J_{i,j}Z_iZ_j$ terms for the problem instance. Suppose we have a qubit j with degree $d_G^{(j)} > h_{\max}$, where $h_{\max}$ is the maximum degree for a register element on the hardware graph. In the terminology of the main paper, $h_{\max} = d_H$ for the hexagon, square, and triangular hardware lattices, while for the heavy-hexagon it is the maximum number of connections per register element $h_{\max} = 3$. If $d_G^{(j)} > h_{\max}$, then at least one SWAP will be needed to enable j to interact with additional qubits, since not all of its interactions can be realized directly on the hardware lattice. In one case, the SWAP could change places of a qubit j' adjacent to j and another qubit j'' that is twice-removed from j such that j can interact with j''. This allows for one additional interaction with j. In a second case, the SWAP gate can switch places of j and an adjacent qubit j', which allows up to $h_{\max} - 1$ new connections between j and j'', j''', etc. So the greatest number of new interactions with j that can be enabled by a SWAP gate is $h_{\max} - 1$. The minimum number of SWAP gates that must be performed for a qubit j to allow it to interact with all adjacent vertices in the cost graph is then

$$N_{\text{SWAP},j}^{\min} = \left\lceil \frac{\delta^{(j)}}{h_{\max} - 1} \right\rceil \tag{1}$$

where

$$\delta^{(j)} = \begin{cases} d_G^{(j)} - h_{\max} & : \quad d_G^{(j)} > h_{\max} \\ 0 & : \quad \text{otherwise} \end{cases} \tag{2}$$

Each SWAP gate switches the hardware locations of two logical qubits and thus can enable new interactions for two logical qubits, that is, a SWAP_{ij} gate could enable new interactions for both i and j. The minimum total number

of SWAP gates is then half the sum of $N^{\min}_{\mathrm{SWAP},j}$ for the individual qubits,

$$N^{\min}_{\mathrm{SWAP}} = \left\lceil \frac{1}{2}\sum_j N^{\min}_{\mathrm{SWAP},j} \right\rceil = \left\lceil \frac{1}{2}\sum_j \left\lceil \frac{\delta^{(j)}}{h_{\max}-1} \right\rceil \right\rceil . \tag{3}$$

Figure S5 compares the observed SWAP gate counts for the $n = 7$ non-isomorphic graphs to the gate counts from the lower bound. For each of the fixed-degree hardwares (all except heavy-hexagon), the average observed SWAP gate counts are no greater than four above the lower bound. To be clear, the lower bound is not expected to correspond exactly with the results, since it makes the simplistic assumption that every SWAP gate enables the maximum possible number of useful new interactions. Thus we expect the observed gate counts to be higher and take these results as suggesting that SABRE is achieving good performance for these graphs. The agreement is somewhat worse for the heavy-hexagon lattice, which has a mixture of vertices of degree two and three in the hardware graph. For this we simply use $h_{\max} = 3$ in computing the lower bound, which ignores additional SWAPs required by the degree-two vertices, so worse agreement is expected.

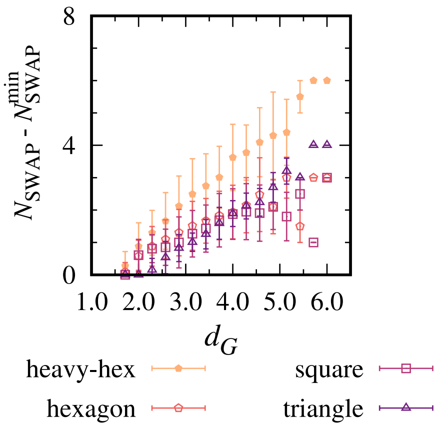

FIG. S5: Difference between observed SWAP gate counts N_{SWAP} and the lower bound $N^{\min}_{\mathrm{SWAP}}$ for 7-vertex graphs.

SWAP scaling with graph diameter

It is interesting to consider how the number of SWAP gates scales with other problem graph properties besides the average degree d_G. We expect that a variety of graph properties that are related to the connectivity of the graph will be correlated with the number of SWAP gates that are needed to implement the graph in a circuit. One such property is the diameter of a graph diam_G, which is the maximum over all minimum distances between pairs of vertices in a graph. When the diameter is small, then the graph is highly connected in the sense that there are short paths between the most distant vertices, while when the diameter is large the graph is less connected in the sense that there is at least one minimal path between vertices that is large. In Fig. S6 we plot the dependence of the number of SWAP gates in terms of $(d_H \mathrm{diam}_G)^{-1}$ for all graphs at $n = 7$ [6]. The N_{SWAP} for each hardware increase linearly with $1/\mathrm{diam}_G$, and the hardware-dependent factor $1/d_H$ gives a unified linear relation for N_{SWAP} across the varying hardwares. Overall, the graph diameter gives an alternate approach to understanding the dependence of N_{SWAP} based on properties of the problem graph, complementary to our analysis in terms of d_G in the main paper.

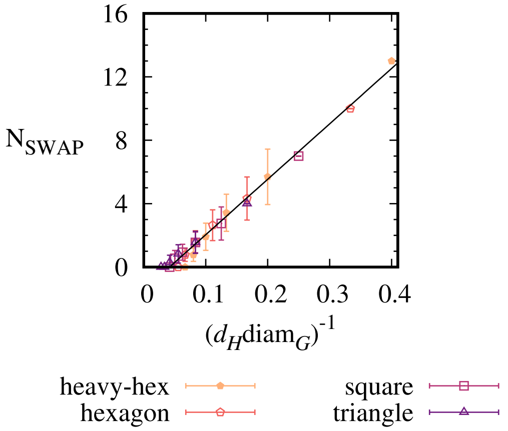

FIG. S6: Difference between observed SWAP gate counts N_{SWAP} and the lower bound $N_{\mathrm{SWAP}}^{\mathrm{min}}$ for 7-vertex graphs.

INITIALLY UNSATISFIED EDGES

Figure S7 shows the average number of two-qubit cost terms $\exp(-i\gamma_l J_{i,j} Z_i Z_j) = \mathrm{CNOT}_{ij}\mathrm{R}_j(2J_{i,j}\gamma)\mathrm{CNOT}_{ij}$ for initially "unsatisfied" problem-graph edges that cannot be implemented directly on the hardware in the initial qubit placement, computed from each of the 3-regular graph test sets, see Methods for details. The number of unsatisfied edges on each hardware scales approximately as $\sim (n - n_0)$ with fit parameters in Table S3. These curves can be united by introducing a scaling factor $1/\sqrt{d_H}$ to each curve, giving the final fit in the Table and Fig. 5 of the main paper.

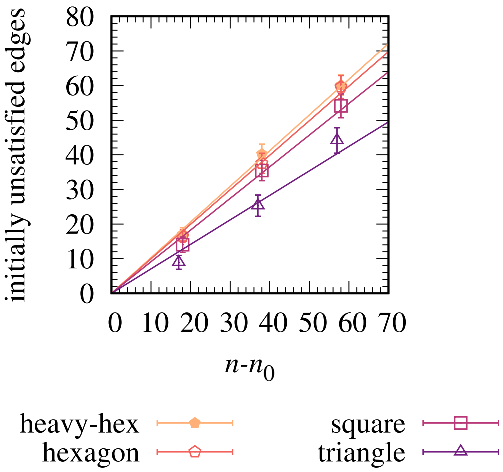

FIG. S7: The number of initially unsatisfied edges for the 3-regular graph test sets on each of the hardwares, with fitting functions and parameters of Table S3.

MEASUREMENT SCALING WITH ERROR RATES, QAOA LAYERS, AND PROBLEM GRAPH DEGREE

Figure S8 shows that the number of measurement samples M to obtain a single measurement from the ideal state distribution increases exponentially with the CNOT gate infidelity ϵ_{CNOT} on fully connected hardware, with $\epsilon_{\mathrm{H}} = \epsilon_{\mathrm{R}} = \epsilon_{\mathrm{CNOT}}/10$ as in the main text. Similar scaling can be observed with increasing numbers of QAOA layers p, since $F_0 \approx f_0^p$ where f_0 is the fidelity lower bound for a single layer. At small F_0 the logarithm $\log(1 - F_0) \approx -f_0^p$, so $M \approx -\log(1 - \mathcal{P})/f_0^p$ and this diverges exponentially in p. Similarly, increasing d_G increases the number of two-qubit edge terms in the QAOA circuit and the fully connected $N_{\mathrm{CNOT}}^{\mathrm{fc}} \sim d_G$. If we further assume the same scaling for

hardware	n_0	fit function	fit parameter
heavy-hex	2	$f_{hh}(n) = \nu_{hh}(n - n_0)$	$\nu_{hh} = 1.03 \pm 0.02$
hexagon	2	$f_h(n) = \nu_h(n - n_0)$	$\nu_h = 0.99 \pm 0.03$
square	2	$f_s(n) = \nu_s(n - n_0)$	$\nu_s = 0.91 \pm 0.04$
triangle	3	$f_t(n) = \nu_t(n - n_0)$	$\nu_t = 0.71 \pm 0.06$
all	2 or 3	$f(n) = \nu(n - n_0)/\sqrt{d_H}$	$\nu = 1.71 \pm 0.04$

TABLE S3: Fit functions and parameter values for the numbers of unsatisfied edges on varying hardware lattices. The final column shows the best fit parameter value and associated asymptotic standard error.

N_{SWAP} as for our $n = 7$ graphs, then $N_{\text{SWAP}} \sim d_G$ and the total number of CNOT gates $N_{\text{CNOT}} \sim d_G$. These factors appear in exponents in F_0 and this gives an exponential divergence in M with respect to d_G.

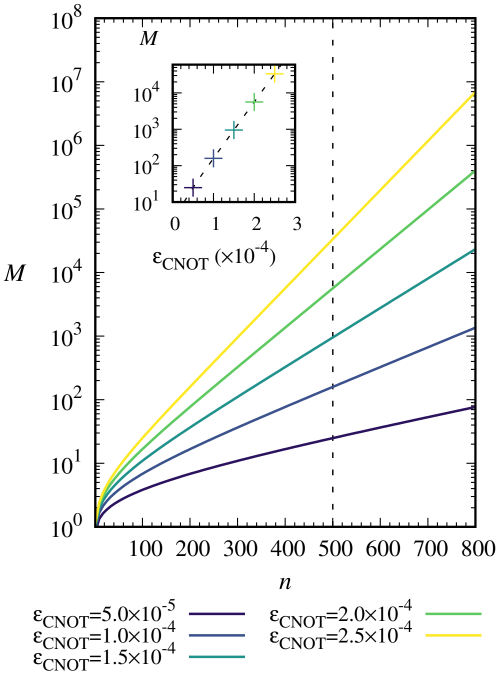

FIG. S8: M scaling on fully connected hardware for varying gate infidelities ϵ_{CNOT} with $\epsilon_{\text{H}} = \epsilon_{\text{R}} = \epsilon_{\text{CNOT}}/10$.

[1] Marcos Yukio Siraichi, Vinícius Fernandes Dos Santos, Caronline Collange, and Fernando Magno Quint ao Pereira. Qubit allocation as a combination of subgraph isomorphism and token swapping. Proc. ACM Program. Lang., 3(OOPSLA, Article 120), 2019.

[2] A. J. McCaskey, E. F. Dumitrescu, D. Liakh, W. Feng, and T. S. Humble. A language and hardware independent approach to quantum–classical computing. SoftwareX, 7:245–254, 2018.

[3] Alexander J. McCaskey, Dmitry I. Lyakh, Eugene F. Dumitrescu, Sarah S. Powers, and Travis S. Humble. XACC: A system-level software infrastructure for heterogeneous quantum-classical computing. arXiv:1911.02452, 2019.

[4] Gushu Li, Yufei Ding, and Yuan Xie. Tackling the qubit mapping problem for NISQ-era quantum devices. arXiv:1809.02573, 2019.

[5] Ritajit Majumdar, Dhiraj Madan, Debasmita Bhoumik, Dhinakaran Vinayagamurthy, Shesha Raghunathan, and Susmita Sur-Kolay. Optimizing ansatz design in QAOA for Max-cut. arXiv:2106.02812v3, 2021.

[6] Rebekah Herrman, Lorna Treffert, James Ostrowski, Phillip C. Lotshaw, Travis S. Humble, and George Siopsis. Impact of graph structures for QAOA on MaxCut. Quant. Inf. Process., 20, 2021.
